# Polymer-assisted enzyme induced carbonate precipitation for non-ammonia emission soil stabilization

**DOI:** 10.1038/s41598-022-12773-6

**Published:** 2022-05-25

**Authors:** Zhen Yan, Sivakumar Gowthaman, Kazunori Nakashima, Satoru Kawasaki

**Affiliations:** 1 Graduate School of Engineering, Hokkaido University, Sapporo, 060-8628 Japan; 2 Faculty of Technology, University of Jaffna, Kilinochchi, 44000 Sri Lanka; 3 Faculty of Engineering, Hokkaido University, Sapporo, 060-8628 Japan

**Keywords:** Civil engineering, Environmental impact

## Abstract

Biocementation using enzyme induced carbonate precipitation (EICP) process has become an innovative method for soil improvement. One of the major limitations in scaling-up of biocement treatment is the emission of gaseous ammonia during the urea hydrolysis, which is environmentally hazardous. In order to eliminate this shortcoming, this paper presents a series of experiments performed to evaluate a novel approach for preventing the ammonia byproducts in the EICP process via the use of polyacrylic acid (PAA). Through the adjustment of the pH to acidic, PAA not only promotes the enzyme activity, but also averts the conversion of ammonium to gaseous ammonia and its release, thus preventing any harm to the environment. The sand samples were treated with cementation solution and assessed for improvement in strength. Calcium carbonate content measurements and X-ray powder diffraction analysis identified the calcite crystals precipitated in the soil pores. Scanning electron microscopy analysis clearly showed that calcium carbonate was precipitated connecting soil particles, thus providing a uniaxial compressive strength (UCS) of up to 1.65 MPa. Overall, the inhibition in the speciation of gaseous ammonia shows the great potential of PAA for large-scale promotion of biocement.

## Introduction

Cement is the most commonly used material in traditional foundation reinforcement, yet its production is substantially energy consuming and environmentally unfriendly. In conventional cement production, the calcium carbonate calcination process not only releases large amounts of CO_2_ but also requires heat of up to 1450 °C during production. The total CO_2_ emissions per tonne of cement produced can be as high as 0.95 tonnes^1^. Thus biological carbonate cement, an important typical biocement, has attracted a lot of attention as cleaner and sustainable biocement ground improvement method that can consolidate loose particles since the early 1990s^2,3^. Extensive laboratory and field investigations have shown that biocement can be widely used in foundation reinforcement^4^, ash settlement^5^, cement crack repairment^6^, slope stabilization^7^ etc.

In microbially induced carbonate precipitation (MICP) and enzyme induced carbonate precipitation (EICP) treatment processes, CaCO_3_ is precipitated in soil pores as the consequence of a set of biological reactions given below in Eqs. (1)–(3). The MICP relies on the ureolytic bacteria, while the EICP process relies on the free urease enzymes usually derived from plants. Regardless of the source of urease, during the exposure of urea, the enzymes catalyst the hydrolysis of urea and produce the carbonates and ammonium (Eq. 1). In the presence of calcium ions, the calcium carbonate precipitates in the soil pores, enabling the cementing bonds between the soil particles^8^. The formation reaction of calcium carbonate is shown in Eq. (2).

1 $$ {\text{CO }} ( {{\text{NH}}_{{2}} } )_{{2}} + {\text{ 2H}}_{{2}} {\text{O}}\mathop{\longrightarrow}\limits^{urease}{\text{CO}}_{{3}}^{{{2} - }} + {\text{ 2NH}}_{{4}}^{ + } $$

2 $$ {\text{CO}}_{{3}}^{{{2} - }} + {\text{ Ca}}^{{{2} + }} \to {\text{CaCO}}_{{3}} \downarrow $$

3 $$ {\text{NH}}_{4}^{ + } + {\text{OH}}^{ - } \leftrightarrow {\text{H}}_{2} {\text{O}} + {\text{NH}}_{3} \uparrow $$

However, the reaction media often becomes relatively alkaline due to the formation of ammonium ions (as per the Eq. (1)), making it conducive for certain amount of ammonium ions produced (up to around 50% at pH 9.24) to be easily converted into ammonia gas, and according to Eq. (3), which is released into the atmosphere. This emission has been an unsolved problem of both the MICP and EICP for decades. Ammonia impose negative impacts on the ecological environment, such as leading to high levels of toxic nitrogen-containing compounds, elevating generation of greenhouse gases^9^, and causing severe injury to health. This limits the application of biocementation technology to large-scale engineering projects.

Thus far, only few alternatives have been proposed for reducing ammonia emission during biocementation process and creating more environment-friendly biocement materials. Notable methods of producing clean bio-cement include the use of asparaginase to drive MICP, which induces 40.6 U/mL of ammonia, significantly lower than that induced by urease (592 U/mL) and achieving a 980 kPa UCS^10^. The use of magnesium phosphate biocements for solidification can reduce ammonia emissions by 75%, and the UCS of over 1.43 MPa could be achieved^11^. The use of bone meal and acid urease to obtain calcium phosphate biocements demonstrates a new economic way of up to 90% ammonia reduction with UCS of up to 1.5 MPa^12^. Thus, finding a cleaner and cheaper way to completely handle the ammonia released during the biosolidification process is still an open requirement.

In this study, an innovative method by using non-toxic polyacrylic acid (PAA) is presented. The PAA in solution kept at a weak acid condition, can not only enhance the acid urease Nagapshin activity, but also keep all the produced ammonia in ion form rather gaseous ammonia. Various characterizations were conducted to examine the samples. These include scanning electron microscopy analyses, X-ray powder diffraction, calcium carbonate content measurement, and estimated UCS values of solidified sand column specimens. The overall performance of this innovative method shows that it is a cleaner and more environmentally friendly approach during full treatment than the conventional MICP/EICP treatment method, laying a solid theoretical foundation for large-scale applications.

## Materials and methods

### Polymer and sand used

**Figure 1:**
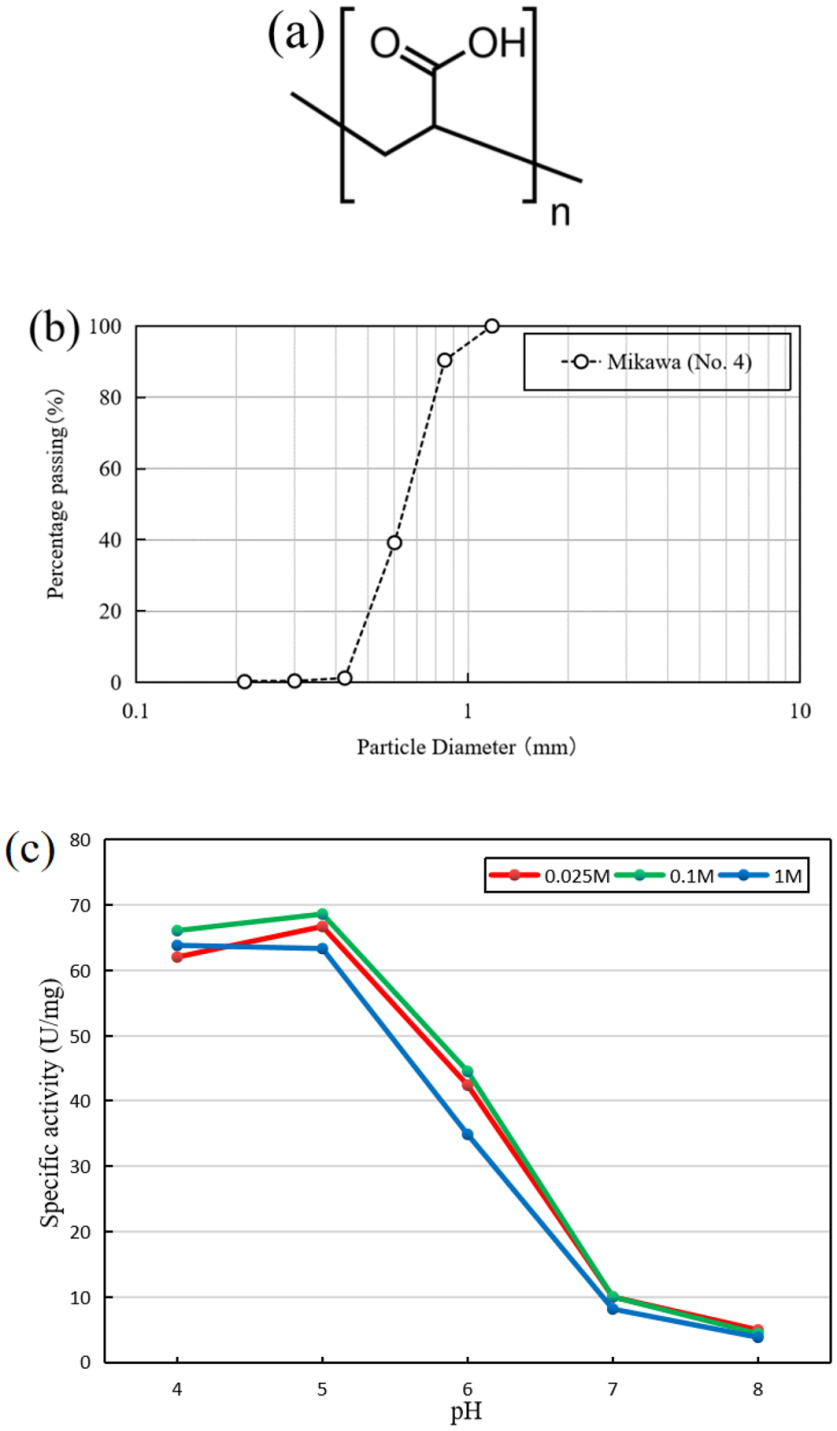
(**a**) The chemical structural formula of PAA used in this study. (**b**) Grain size distribution curve of silica sand used in this study. (**c**) The specific activity of acid urease under different urea concentration in this study^12^.

The PAA herein used for adjusting the solution pH to relatively acidic was obtained from Wako Pure Chemical Industries Ltd., (Tokyo, Japan). The average molecular mass is 25,000. And the chemical structure of the PAA is shown in Fig. 1a.

The sand used in this research work was Mikawa No. 4 sand which is a commercially available silica sand and has been used multiple times in previous studies of biocement^7,13,14^. The maximum and minimum dry densities of the sand are 1.476 and 1.256 g/cm^3^, respectively. The particle density and mean diameter (D_50_) is 2.66 g/cm^3^ and 0.87 mm, respectively. The grain size distribution of Mikawa No. 4 sand is presented in Fig. 1b. According to the Unified Soil Classification System (USCS), this sand can be classified as poorly graded sand^15^. Before the experiments, the sand was oven dried at 90 °C for 48 h to ensure complete drying.

### Acid enzyme and PAA tolerance tests

The acid urease (from *Lactobacillus fermentum*), Nagapshin, used herein was obtained from NAGASE and Co., Ltd. (Tokyo, Japan). As shown in the Fig. 1c^12^, the specific activity of the enzyme is much higher under acidic conditions than neutral and basic^16^. It provides a strong support for our intention to suppress the production of ammonia under acidic conditions.

To determine whether the acid urease is tolerant to PAA, in other words, whether it can maintain high activity despite the acidic conditions provided by PAA, a series of test tube tests were designed for investigation. And the experimental conditions are shown in Table 1 together with the precipitation tests. The pH of the solution was measured before and after the tests, by using LAQUA-9615S pH meter, HORIBA Advanced Co., Ltd., Japan. Considering that the majority of cases are acidic to neutral, the measurement of the post-experimental concentration of ammonium ions was chosen to evaluate the effect of PAA concentration on the acid urease activity over the concentration of carbonate ions.

**Table 1:** Experimental conditions for PAA tolerance tests and precipitation tests.

PAA (g/L)	0 M Reagent (CaCl_2_-Urea), acid urease 5 g/L	0.25 M Reagent (CaCl_2_-Urea), acid urease 5 g/L	0.5 M Reagent (CaCl_2_-Urea), acid urease 5 g/L	0.75 M Reagent (CaCl_2_-Urea), acid urease 5 g/L	1.0 M Reagent (CaCl_2_-Urea), acid urease 5 g/L	0.5 M Urea, acid urease 5 g/L
0	A1	B1	C1	D1	E1	F1
5	A2	B2	C2	D2	E2	F2
10	A3	B3	C3	D3	E3	F3
15	A4	B4	C4	D4	E4	F4
20	A5	B5	C5	D5	E5	F5
25	A6	B6	C6	D6	E6	F6
30	A7	B7	C7	D7	E7	F7

The ammonium ions were measured using indophenol spectrophotometry method^17,18^. In the presence of hypochlorite, ammonium ions from the hydrolysis of urea react with phenol to produce a blue indigo dye in an alkaline medium, the intensity of which is measured at the wavelength of 630 nm (OD_630_) by UV–visible spectroscopy, V-730, JASCO Corporation, Tokyo, Japan. Ammonium concentrations were obtained through the establishment of calibration curves between different ammonium ion concentrations and intensities (OD_630_).

This method has been applied multiple times to quantify the concentration of ammonium ions produced by urea hydrolysis, and the enzyme activity can be accurately derived per unit time. However, in this experiment, 24 h were chosen to determine the effect of different concentrations of PAA (gradually increasing from 0 to 30 g/L) of the acid enzyme on the enzyme's ability to hydrolyze urea in the same 10 mL solution conditions (25 °C, 0.5 M urea).

### Precipitation test tube tests

To investigate the precipitation tendency of CaCO_3_, a series of experiments were performed with different combinations of reagent (CaCl_2_, urea) and the same amount of urease at different PAA concentrations. This set of experiments was carried out in transparent tubes. 10 mL of the solution of the reaction mixture of CaCl_2_, urea and acid urease were formulated as summarized in Table 1. Keeping it all at 25 °C and 160 rpm in a shaker for 24 h, and the pH of the solution was measured after the reaction. After the reaction, the concentrations of calcium ions and ammonium ions were also measured to assess the progress of the reaction. Calcium ions were measured using a LAQUA-twin calcium meter, HORIBA Advanced Techno Co., Ltd., Japan. Ammonium ions were also measured by indophenol spectrophotometry.

The samples were centrifuged to separate the CaCO_3_ precipitate from the supernatant, and the precipitates were dried in an oven at 60 °C for 24 h. The dry weights of the precipitates were determined as *W1*. This was followed by rinsing with analytical grade 3 M hydrochloric acid, and after all the calcium carbonate in the precipitate has reacted, the centrifugation and drying were repeated. The recorded weight at this stage was *W2*. As given in Eq. (4), the difference between *W1* minus *W2* is the calcium carbonate precipitated. The precipitation test tube tests conditions are shown in Table 1, in which all the cases contained acid enzyme at the concentration of 5 g/L.

4 $$ {\text{CaCO}}_{{3}} \;{\text{precipitation}}\;{\text{weight}}\;( {\text{g}} )  = W1 - W2 $$

### Lab level solidification tests

Sand solidification tests were performed using a 50 mL syringe as a model (30 mm in diameter and 70 mm in height) to check the effect in the presence of the same concentration of urea and calcium chloride and different concentrations of PAA. The solidification tests conditions were the same as for the C1 to C4 groups in the precipitation tests, with 0.5 M of both urea and calcium chloride, as shown in Table 2, named as SC1 to SC4.

**Table 2:** Experimental conditions for solidification tests.

Case no	CaCl_2_ (mol/L)	Urea (mol/L)	PAA (g/L)	Acid urease (g/L)
SC1	0.5	0.5	0	5
SC2	0.5	0.5	5	5
SC3	0.5	0.5	10	5
SC4	0.5	0.5	15	5

**Figure 2:**
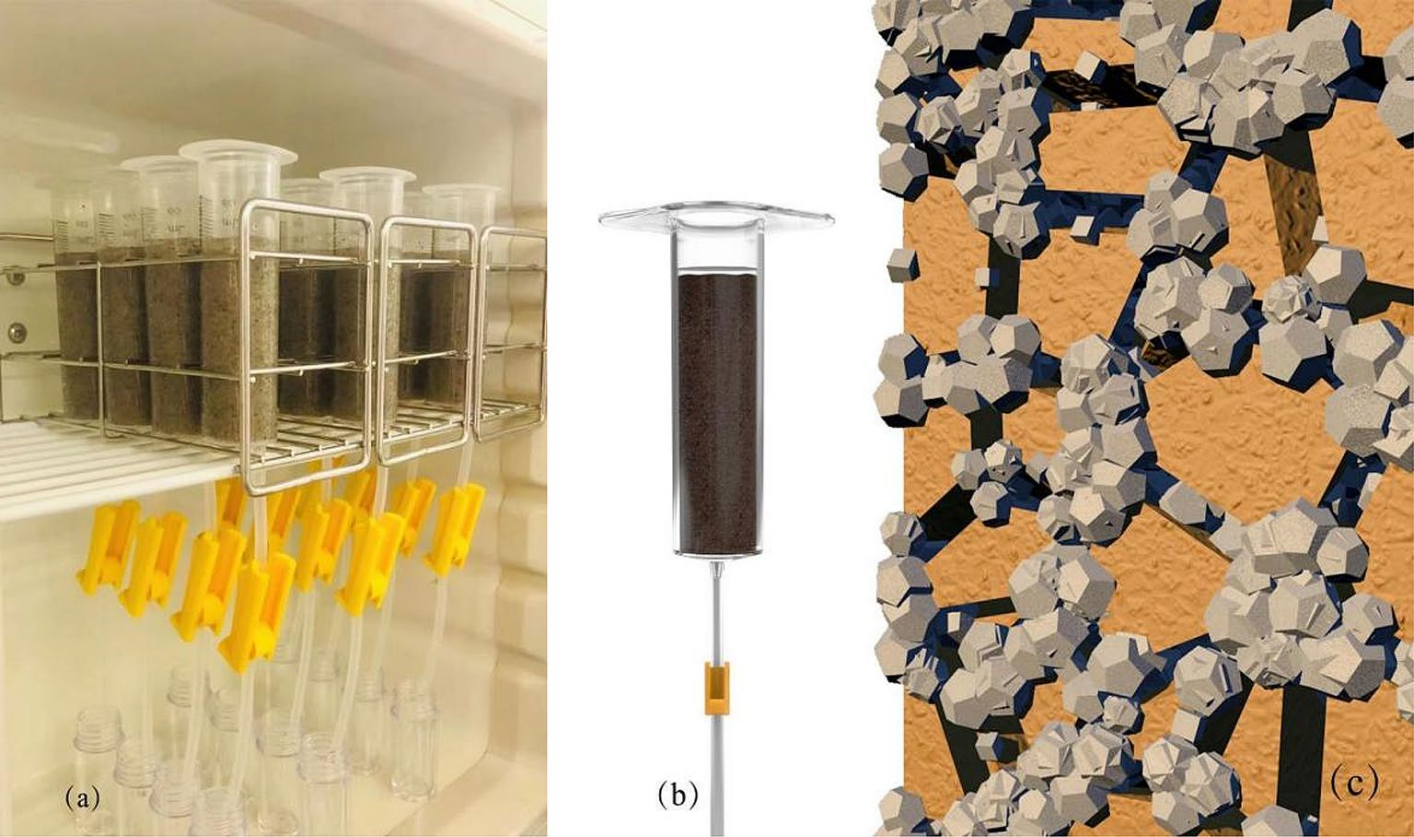
The experimental set up of sand specimens: (**a**) photo of all samples, (**b**) diagram of individual specimen, (**c**) mechanism of solidification schematic.

Each case was prepared with 70 g Mikawa (No. 4) sand, placed into a syringe in three layers. Each layer was subjected to 20 hammer blows. This method was also used previously by many researchers for small size specimens, and the experimental setup is illustrated in Fig. 2a,b and the mechanism of solidification is shown in Fig. 2c.

The components other than Nagapshin acid urease were configured in advance as a pre-cementation solution. At each injection, the acid enzyme was then mixed with 20 mL of the pre-cementation solution. After the enzyme was fully dissolved, the cementation solution was immediately injected into the mold and adjusted to ensure that 1 mm of the solution was left above the surface to enable the grout solution to reach the entire sand column uniformly and remain there for 24 h. For all injections throughout the whole treatment time, the cementation solution is injected by gravity only.

The experiments were performed in an incubator at 25 °C in triplicate, 14 times over two weeks. Each day, the former cementation solution was drained and the injection operation described above was repeated to inject new solution. The pH, calcium ion concentration and ammonium ion concentration of the effluent were measured after each treatment. After 14 days of curing, the specimens were sufficiently rinsed using distilled water prior to further experimentations, which is mainly to eliminate the unreacted/soluble chemicals. Then the syringe molds were cut, and the specimens were carefully removed from the molds. The biocementation strength of the specimens was examined with the soft rock penetrometer (SH-70, Maruto Testing Machine Company, Tokyo, Japan), and the UCS was estimated from the NP according to following regression equation.

5 $$ {\text{log}} ( y )  = 0.978{\text{ log}} ( x )  + 2.621 $$

where the correlation coefficient is 0.941, *x* is NP (N/mm), and *y* is UCS (MPa). As mentioned in the instrument manual, this calibration equation was developed by considering 114 natural rock samples and 50 soils amended with cement. And this method has already been widely used for reliable evaluation of the UCS of the biocemented soil specimens^14,19^.

### Determination of the biocement content

The precipitated CaCO_3_ content of the treated specimens was measured by the ASTM (ASTM D4373-14) stipulated method for the determination of carbonate content of soils and soft rock^20–22^. The standard is based on the linear relationship between the mass of calcium carbonate and CO_2_ pressure induced by the reaction between CaCO_3_ and hydrochloric acid (HCl). The standard apparatus includes a reaction cylinder, small cups filled with HCl and a pressure gauge. A pre-weighed sand sample was placed inside the reaction cylinder along with cups filled with HCl and closed tightly to prevent any gas leakage. The cylinder was tilted so that acid reacts with the treated sand and shaken until a constant gas pressure was achieved. Finally, the calcium carbonate content was determined through a pre-calibrated curve made using analytical grade CaCO_3_ powder.

### Mineralogical and morphological inspection

The chemical components of the sand columns were determined by X-ray diffraction (XRD; MiniFlex, Rigaku Co., Ltd., Tokyo, Japan) analysis for the ground samples under Ni-filtered Cu 1.5406 Å radiation at a rate of 6.5° 2*θ*/min ranging from 5° to 70° 2*θ*. Qualitative mineralogy of the samples was determined with the standard interpretation procedures of XRD using a software for phase identification from powder diffraction. Scanning electron microscopy (SEM; Miniscope TM3000, Hitachi, Tokyo, Japan) was used to investigate the morphologies of the precipitated crystals inside of the sand columns.

## Results and discussion

### PAA tolerance tests

**Figure 3:**
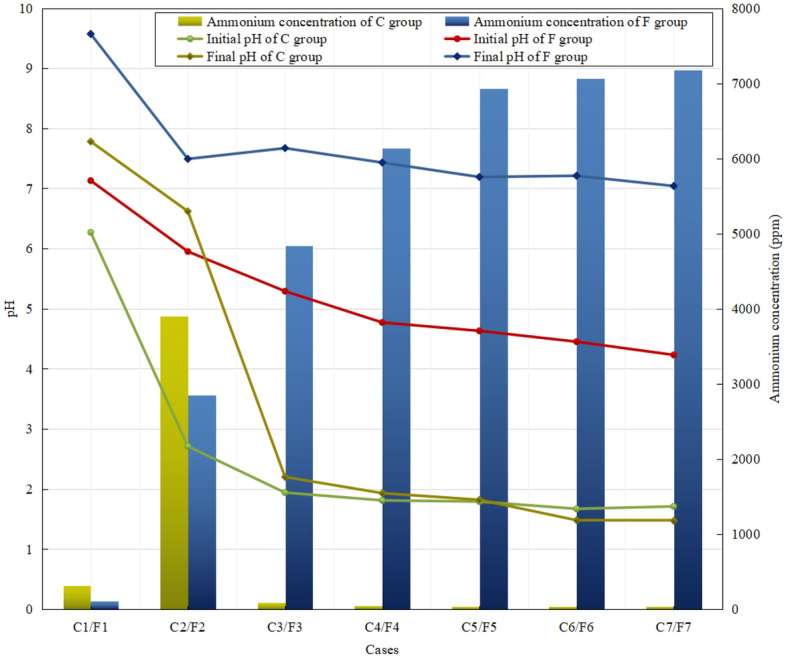
The pH and the concentrations of NH_4_^+^ ions in group C and group F.

With the assistance of the calibration curve between ammonium ion concentration and OD_630_, obtained the concentration of ammonium ion after 24 h using this method. It can be seen from Fig. 3 that the ammonium ion concentration increases significantly with the increase of PAA concentration. Combined with the analysis of pH, we can know that PAA only affects the hydrolysis ability of the enzyme through pH control. When PAA is 0 g/L, the solution pH is neutral, and the activity of acidic enzyme is low at this time, and the ammonium obtained by hydrolysis is very little. When PAA gradually increases, the pH of the solution becomes lower and lower, the enzyme activity is improved, and the ammonium obtained by hydrolysis increases sharply. It is noteworthy that, combined with Fig. 1c, the enzyme also has a certain amount of activity in a neutral environment, but the resulting ammonium ion concentration is close to 0 ppm. This indicates that the final pH has a significant effect on the concentration of ammonium ions. From the final pH greater than 9, it can be seen that the pH is close to the equilibrium point, so nearly half of the ammonium ions were converted to ammonia and released. However, since the ammonium ion content of F2 and other groups is statistically significant and greater than the value of F1, the conclusion reached is that the PAA only affects the enzyme hydrolysis ability through holding of pH.

### Observations of precipitation tests

In order to show more clearly the effect of PAA concentration and reagent (calcium chloride and urea) concentration on calcium carbonate precipitation, two three-dimensional graphs are used to show the results.

**Figure 4:**
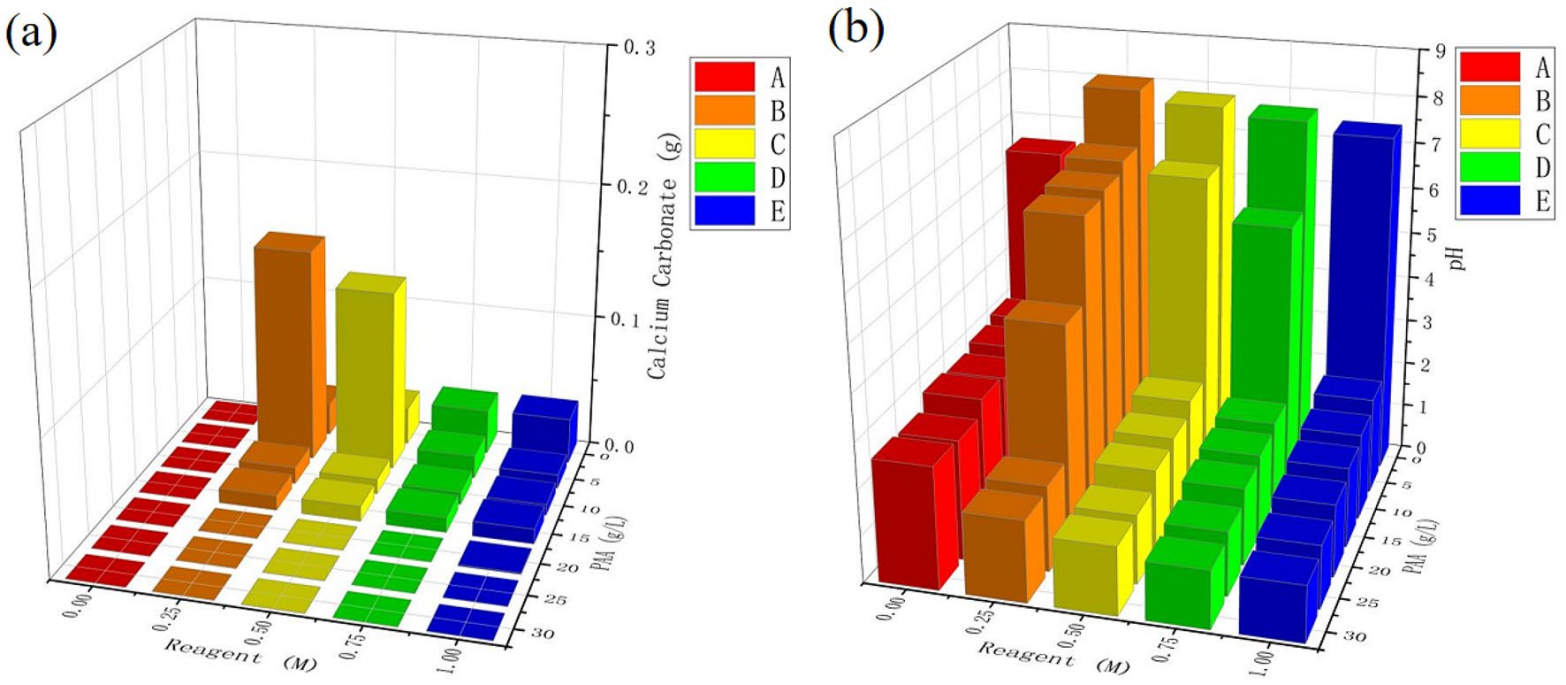
Precipitation tests results: (**a**) amount of calcium carbonate precipitation, (**b**) final pH.

It can be seen from the Fig. 4a that the mass of precipitated calcium carbonate first increased and then decreased, when the PAA concentration is kept constant (especially when PAA is less than 15 g/L), and the reagents are slowly transitioned from 0 to 1 M. This is because the hydrolytic capacity of the enzyme is affected by the urea concentration despite being at the same concentration of PAA. As shown in Fig. 1c, too high a concentration of urea, on the contrary, slightly inhibits the enzymatic activity and thus affects the precipitation of calcium carbonate. It should be noted that the urea is a chaotropic agent, which destabilizes the urease (protein) at their high concentrations, i.e., the hydrophobic interactions in urease molecules are disrupted, resulting in reduced functionality of enzyme. This could possibly be attributed to the different trend observed for the concentrations of above 0.5 M.

It is worth noting that in Fig. 4b when combining groups F and C, we can see that the initial pH of group F is higher than the final pH of group C. This is due to the presence of calcium chloride in group C, which is weakly acidic.

When the reagent is 0.25 M and 0.5 M, the calcium carbonate shows a bell-shaped curve with the increase of PAA and reaches the maximum precipitation at 5 g/L of PAA. When PAA was greater than 15 g/L, the precipitation amount was close to 0 g. This is because the enzyme has high activity under acidic conditions, but excess PAA inhibits the precipitation of calcium carbonate (too acidic). This finding also helped us to design the conditions for the coagulation experiments, and the concentration range of PAA is reduced to less than 15 g/L PAA.

The comprehensive analysis in combination with the pictures illustrates the reaction in two stages for better understanding, although the two stages actually proceed simultaneously. In the first stage, the addition of PAA causes the pH of the solution to decrease, thus increasing the hydrolysis capacity of the enzyme. The second stage is that the enzyme hydrolyzes the urea into ammonium and carbonate ions while raising the pH of the solution, so that when the final pH after these two stages is still relatively acidic or neutral, and the precipitation of calcium carbonate will only continue at this point. If the final pH is too acidic due to the excessive addition of PAA, then the precipitation of carbonate will be greatly limited. This also provides a theoretical basis for controlling ammonia emissions. If the final pH can be controlled to be relatively acidic, then ammonia emissions will be suppressed without inhibiting the precipitation of calcium carbonate.

### Observations during the solidification treatment

**Figure 5:**
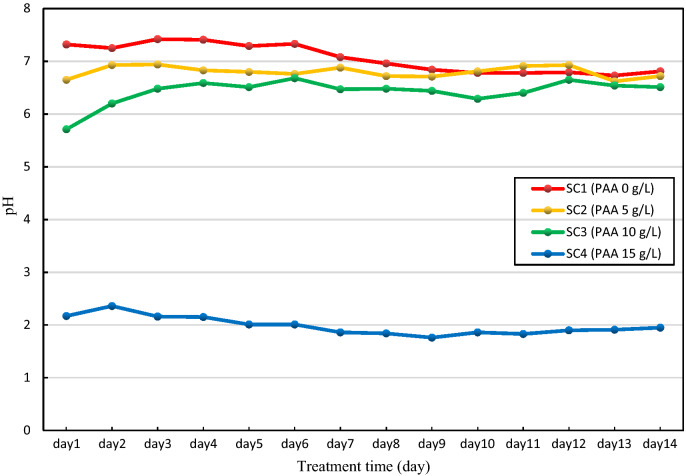
The pH in effluent of the specimens treated by cementation solution with different PAA concentration.

As shown in Fig. 5, the pH of SC1 effluent is slightly alkaline at the beginning and even slightly acidic at the end of the process. It is worth noting that the effluent of SC2, SC3 and SC4 are all less than 7 in the whole process.

The pH of SC1 decreased slightly in the later days, while the pH of SC2 and SC3 increased slightly in the later days. This is due to the fact that the process of configuring the cementation solution is determined in steps rather than configuring a new one daily. The solution was first configured as pre-cementation solution and bottled then stored, while 20 mL of pre-cementation solution was poured before each experiment and the enzyme was added and left to dissolve before use. Therefore, the pre-cementation dissolves the carbon dioxide in air when exposed, and the carbonic acid produced causes a slight decrease in the pH of the pre-cementation solution, which in turn causes a decrease in the pH of the cementation solution.

From the analysis of the two-stage mechanism described previously, for SC1, the pH drop in the first stage leads to a slight increase in enzyme activity, but it is still at a relatively low state (because enzymes are not too sensitive to pH changes in a neutral environment). In the second stage, the urea is hydrolyzed into ammonium and carbonate ions and the pH is increased. Since the solution already has carbonic acid from dissolved carbon dioxide in the air and carbonate from ionization, the reaction at this stage is actually inhibited, which in turn leads to a decrease in the pH of the effluent at a later period. For SC3, the carbonic acid produced by the dissolved carbon dioxide promotes the enzyme activity in the first stage. In the second stage, more urea is hydrolyzed by the enzyme. Ammonium and carbonate are produced, and the pH of the later effluent is increased. As for SC4, as in the precipitation experiments, the pH was maintained at acidic conditions, which was highly detrimental to the precipitation of calcium carbonate.

### Strength and uniformity of biocemented sand columns

**Figure 6:**
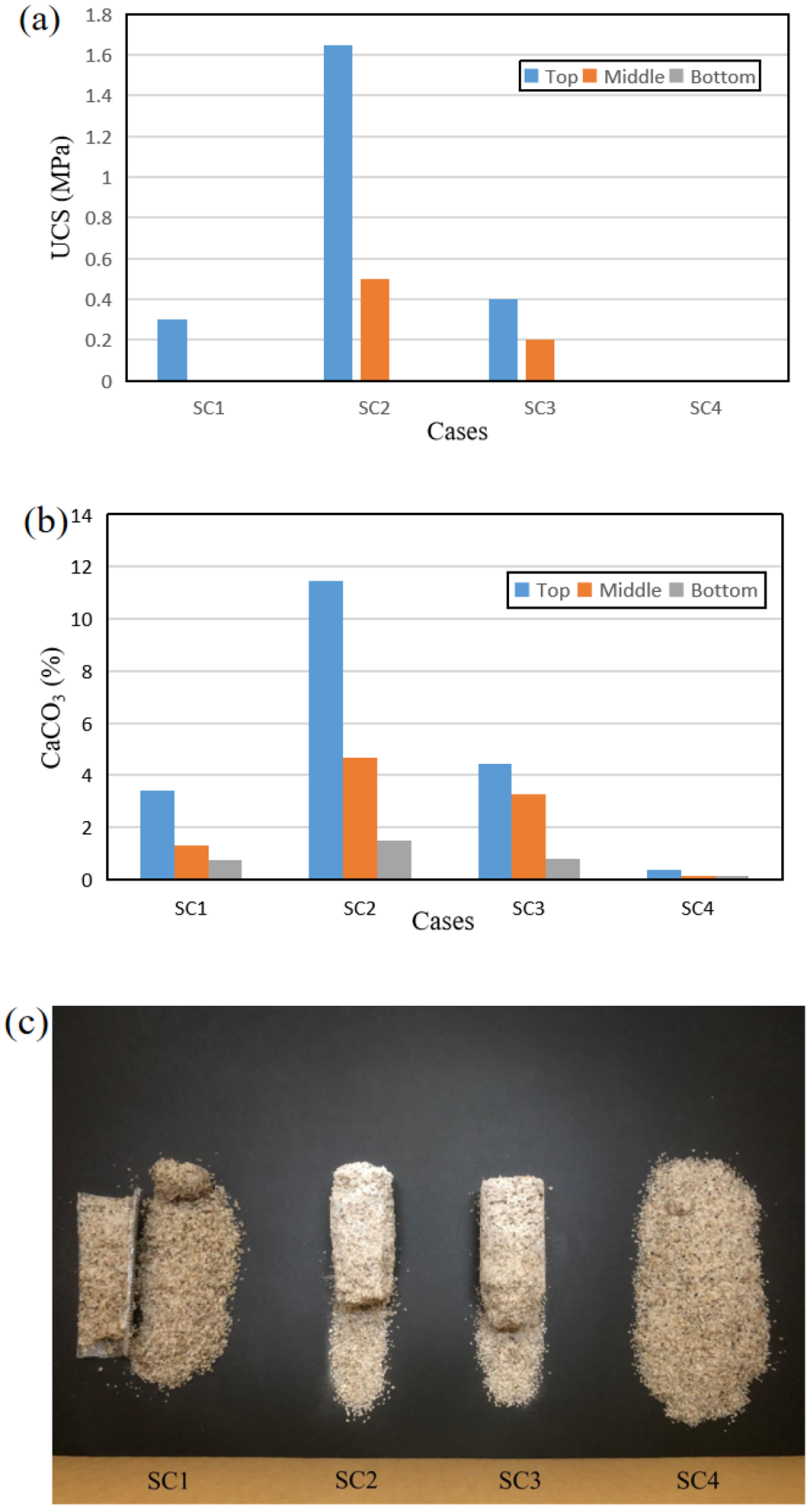
(**a**) Comparison of the estimated UCS of specimens, (**b**) the precipitation contents of calcium carbonate in treated specimens, and (**c**) the physical appearance of the specimens.

From the results obtained, SC2 has the highest UCS intensity, while SC4 has no measurable UCS strength at all. Moreover, by combining Fig. 6a,c, the samples from top to bottom showed a trend that the UCS was the strongest at the top and the lowest at the bottom. According to the previously proposed classification system, all sand samples can be classified as strongly cemented.

Combining Fig. 6b and analysis results of the content in the sand column measured by the calcium carbonate detector, there is a direct correlation between the amount of precipitated CaCO_3_ and the estimated UCS values from SC1 to SC4, and from the top to the bottom in a single sand column.

For the explanation of the maximum UCS strength in SC2, it is also evident from the conclusions obtained from previous precipitation tests that a level of PAA of 5 g/L is the most suitable group for calcium carbonate precipitation among these groups. It does not constitute an inhibition, so this group has the most calcium carbonate content and the largest estimated UCS value. The explanation for the strength of SC1 and SC4 is that SC1 is explained by the fact that the enzyme activity was not promoted in any way in the first stage, while SC4 is explained by the fact that the acidic conditions were maintained despite the presence of urea hydrolysis after the second stage due to the excess of PAA, which greatly inhibited the precipitation of calcium carbonate.

Interestingly, the strength of the sand columns gradually decreased from top to bottom which was the result of uneven distribution of calcium carbonate, probably due to bio-blockage. Calcium carbonate at the top is the first to precipitate, resulting in smaller pores between sand grains, which leads to a decrease in precipitation at the bottom as chemical transport (cementation solution) through the pores receives obstruction, ultimately manifesting as a gradual decrease in the strength of the UCS from top to bottom^7^. However, this aspect has not been adequately studied, and it is difficult to give a clear explanation; therefore, further studies are needed.

### Characteristics of precipitated biocement in sand columns

The morphology of the microstructure was studied by analyzing SEM images of the specimens of the soil solidification tests shown at the same scale. It can be seen that in the absence of any polymer, membranous calcium carbonate adheres to the surface of the sand grains. When PAA was added to the medium at 5 g/L, web-like formation was seen on the surface of the sand grains, and the sand grains were interconnected. With further increase of PAA concentration, the number and size of calcium carbonate precipitates decreased and there was almost no connection between sand grains. The amount of calcium carbonate in SEM photographs also again verified the results of precipitation tests.

**Figure 7:**
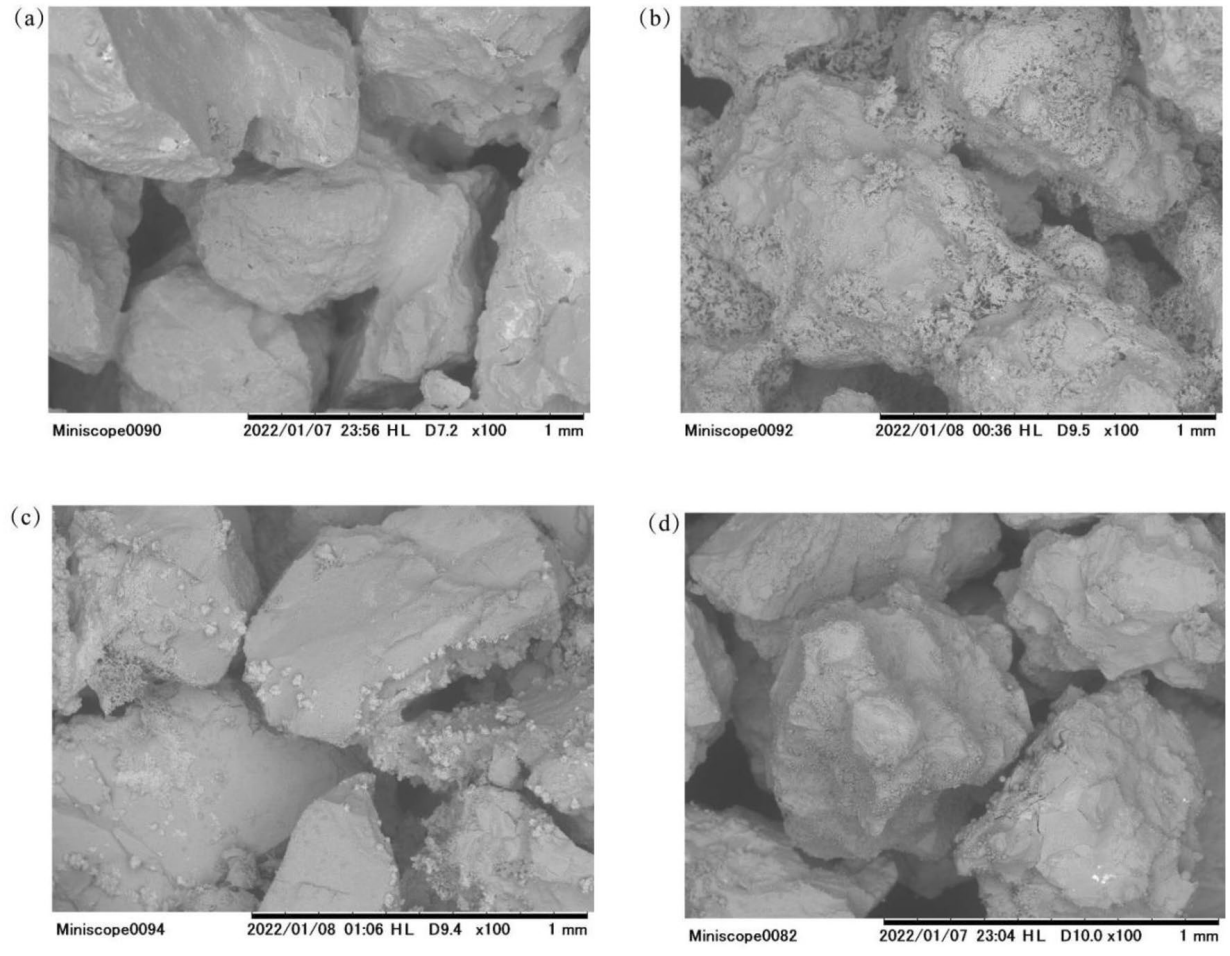
The SEM images of the treated sand matrix for the (**a**) PAA = 0 g/L (Case SC1), (**b**) PAA = 5 g/L (Case SC2), (**c**) PAA = 10 g/L (Case SC3), (**d**) PAA = 15 g/L (Case SC4).

It can be seen from the SEM images in Fig. 7b that SC2 forms a strong bond between the sand grains, and this microstructure creates an irregular interlocking and enhances the stability of the bio-sandstone. The increase of uniaxial compressive strength up to 1.65 MPa also proves this.

In contrast, Fig. 7d shows a small number of calcium carbonate crystals in the sand particles and their small size. While SC1 in Fig. 7a and SC3 Fig. 7c are in between the two extremes, with only calcium carbonate crystals as partially connected soil particles.

**Figure 8:**
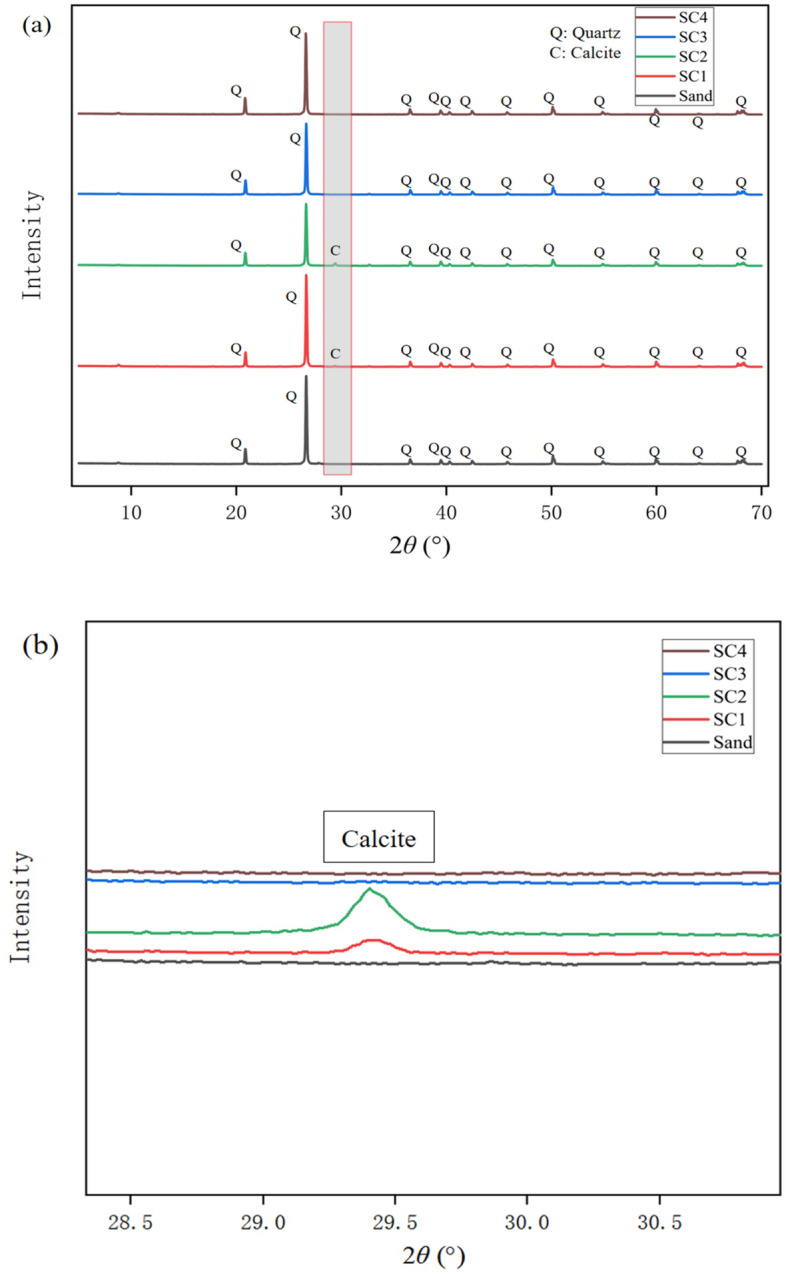
The XRD analysis for the test cases (**a**) overall comparison, (**b**) partial enlargement comparison.

XRD was used to analyze the chemical composition of the sand columns cemented with bio-cement, as well as the Mikawa No. 4 sand, and the strength distribution is shown in Fig. 8. The XRD raw data obtained was analyzed using the software program, MATCH. From the results, there is no doubt that SC1 and SC2 have calcite peaks near 2*θ* of 29°, which is one of the signs of success in this experiment. Although SC3 and SC4 do not have calcite peaks in XRD, considering the SEM pictures, perhaps due to the extremely small size of calcite produced by precipitation, XRD was not able to analyze the extremely small size samples properly.

## Conclusion

In this study, a new eco-friendly CaCO_3_ biofortification method was proposed and demonstrated in a laboratory scale for soil improvement. The strategy involves a two-stage pH-dependent mechanism in which the first stage controls the initial pH to be acidic through PAA, increasing the hydrolysis capacity of acidic enzymes, and the second stage increases the pH from acidic to relatively acidic levels through the urea hydrolysis process, enabling the precipitation of calcium carbonate. Injection of a colloidal solution consisting of PAA, calcium chloride, urea and acid urease showed the formation of insoluble calcium carbonate colloids that bound the soil particles together.

The pH increase during the reaction could be effectively controlled by the PAA content in the cementing solution. The results showed that the increase in pH had a significant effect on (i) the amount of calcium carbonate precipitation and (ii) the morphology of the formed crystals, indicating that care should be taken to effectively select the PAA concentration in the CaCO_3_ cementing solution. XRD analysis showed that the formed crystals were all calcite. However, at a PAA concentration of 5 g/L in the cementing solution, platelet crystals were more readily formed in the sand matrix and the uniaxial compressive strength increased to 1.65 MPa.

An important aspect of this approach is the complete elimination of gaseous ammonia emissions. In a typical MICP/EICP biocement, approximately 5.8 g of gaseous ammonia is emitted for every 1 L of solidification fluid injected. In fact, gaseous ammonia emissions are much more difficult to suppress and much less easily controlled. Since the pH of the effluent in the sand coagulation test can be easily controlled below neutral conditions, the release of toxic gaseous ammonia can be completely eliminated (up to 100% compared to conventional biocements).

This is due to the complete control of gaseous ammonia release compared to typical MICP/EICP biocements, thus showing the great economic and ecological advantages of the method. The proposed method provides a new direction for greener and more environmentally friendly soil improvement based on bio-cement. With these important findings, further research is encouraged to facilitate the implementation of this eco-friendly method in the field.

## Data Availability

All the experimental data that support the findings of this study are available from the corresponding author upon reasonable request through email.
